# Effects of Benzalkonium Chloride on THP-1 Differentiated Macrophages *In Vitro*

**DOI:** 10.1371/journal.pone.0072459

**Published:** 2013-08-19

**Authors:** Sylvain Michée, Françoise Brignole-Baudouin, Luisa Riancho, William Rostene, Christophe Baudouin, Antoine Labbé

**Affiliations:** 1 INSERM, U968, Paris, France; 2 UPMC University Paris 06, UMR S 968, Institut de la Vision, Paris, France; 3 CNRS, UMR 7210, Paris, France; 4 Centre Hospitalier National d′Ophtalmologie des Quinze-Vingts, INSERM-DHOS CIC 503, Paris, France; 5 Université Versailles Saint-Quentin-en-Yvelines, Versailles, France; 6 Université Paris Descartes, Sorbonne Paris Cité, Faculté des Sciences Pharmaceutiques et Biologiques, Paris, France; 7 Assistance Publique - Hôpitaux de Paris Hôpital Ambroise Paré, Service d′Ophtalmologie, Boulogne-Billancourt, France; National Institute of Infectious Diseases, Japan

## Abstract

**Purpose:**

To characterize the effects of benzalkonium chloride (BAK) in THP-1 differentiated cells *in vitro*.

**Methods:**

Macrophages were obtained after differentiation of THP-1 cells, a human monocytic leukemia cell line. Macrophages were exposed for 24 h to 33 nM (10^−5^%) benzalkonium chloride (BAK), 10 nM dinitrochlorobenzene (DNCB), 100 ng/mL lipopolysaccharide (LPS), 5 ng/mL tumor necrosis factor alpha (TNF-α) or phosphate buffered saline (PBS) as controls. The expression of CD11b, CD11c, CD33 and CD54 was evaluated using immunohistochemistry and flow cytometry (FCM). Phagocytosis function was analyzed using carboxylate-modified fluorescent microspheres and quantified by FCM. Migration was evaluated in cocultures with conjunctival epithelial cells. Cytokine production was detected and quantified in culture supernatants using a human cytokine array.

**Results:**

Stimulation of THP-1-derived macrophages with a low concentration of BAK increased CD11b and CD11c expression and decreased CD33. Macrophages exposed to BAK, LPS and TNF-α had increased phagocytosis. In contrast to LPS, BAK and TNF-α increased macrophage migration. Cytokines in supernatants of macrophages exposed to BAK revealed an increased release of CCL1, CCL4/MIP-1β, TNF-α, soluble CD54/ICAM-1 and IL-1β.

**Conclusion:**

*In vitro*, BAK has a direct stimulating effect on macrophages, increasing phagocytosis, cytokine release, migration and expression of CD11b and CD11c. Long-term exposure to low concentrations of BAK should be considered as a stimulating factor responsible for inflammation through macrophage activation.

## Introduction

Inflammatory and immune cells are involved in the pathophysiological mechanisms of ocular surface diseases [1]. Macrophages are mononuclear phagocytes, playing a pivotal role in tissue homeostasis as scavenger cells silently removing dead and dying epithelial cells, and facilitating regrowth of injured tissues [2]. Alongside dendritic cells and B-lymphocytes, they are antigen-presenting cells [2]. They produce inflammatory mediators and exhibit heterogeneity in their phenotype depending on the local environment. In the conjunctiva, macrophages are localized in the stroma and can interact closely with conjunctival epithelial cells, playing an important role in ocular surface diseases. For example, allergic conjunctivitis has been shown to be associated with excessive macrophage infiltration in the conjunctiva [3], [4]. In trachoma, they are known to increase local production of connective tissue growth factor (CTGF), basic fibroblast growth factor (bFGF), and vascular endothelial growth factor (VEGF) [5]. Similarly, in ocular cicatricial pemphigoid, significant infiltration of macrophages was observed in the conjunctiva [6].

Due to the subclinical inflammation caused by long-term use of antiglaucoma eyedrops, patients treated for glaucoma or ocular hypertension frequently suffer from ocular surface disease [7]. Preservatives associated with the active compound in eyedrops have already shown their toxic effects in clinical and experimental studies, and could be, at least in part, responsible for such inflammatory changes [7]. Benzalkonium chloride (BAK), a quaternary ammonium, is the most commonly used preservative in antiglaucoma medications [7]. BAK is directly responsible for goblet cell loss, conjunctival squamous metaplasia and apoptosis, and disruption of the corneal epithelium barrier [8]. It induces a dose-dependent cytotoxicity leading to apoptosis and necrosis of conjunctival cells [9]. In glaucomatous patients, an association between ocular surface disease severity and the number of BAK-containing eye drops has been described [7], [10]. Similarly, Malvitte *et al*. showed that the expression of fibroblastic and inflammatory markers as well as the intensity of the inflammatory reaction seemed to be related to the number of preservative-containing medications used and the duration of treatment [11]. Increased amounts of inflammatory cells, especially macrophages, within the stroma have been observed following long-term applications of preserved antiglaucoma eyedrops [12], [13]. Such inflammatory processes stimulate postoperative conjunctival fibrosis at the filtration site and are the principal cause of failure of glaucoma filtering surgery [14]. In comparison with patients treated with unpreserved eyedrops, conjunctival biopsies from patients treated with preserved eyedrops containing BAK presented increased macrophage tissue infiltration [15]. Similarly, in an animal model comparing instillations of timolol associated with BAK versus unpreserved timolol, conjunctival biopsies showed an increased density of macrophages in animals exposed to BAK [15]. Beside the prominent role of macrophages in ocular surface disease, inflammatory cells have also been found in trabecular meshwork in primary angle-closure glaucoma [16], as well as playing a role in trabecular degeneration in primary open angle glaucoma, especially in patients receiving multiple therapy over the long term [15]. As BAK has been recently found in the trabecular meshwork after prolonged instillation even in healthy eyes in rabbit model [17] it can be hypothesized that BAK stimulates macrophage infiltration and interact with inflammatory cells within the trabecular meshwork.

The effects of BAK on THP-1 cells have already been investigated for toxicological and allergic purpose [18], [19]. However, THP-1 cells are monocytic cells, namely circulating blood cells, and interaction with BAK cannot fully reflect the effects of this toxic on cells in tissues where monocytes differentiate into macrophages. Therefore, considering the pivotal role of macrophages in ocular surface diseases and particularly in patients treated for glaucoma or ocular hypertension, the objective of the study was to characterize the effects of BAK on THP-1 derived macrophages *in vitro*, especially on their activation phenotype, cytokine production, migration and phagocytosis functions.

## Materials and Methods

### Cell lines

Macrophages were obtained after differentiation of THP-1 cells provided by the American Type Culture Collection (TIB-202™, ATCC, Rockville, MD, USA). This cell line comes from acute monocytic leukemia. THP-1 cells are known to differentiate into macrophages after stimulation with phorbol myristate acetate (PMA) [20]. Cells were cultured in RPMI 1640 (Roswell Park Memorial Institute) (Invitrogen Life Technologies, Paisley, Scotland, UK) supplemented with 10% fetal bovine serum (HyClone, Logan, UT, USA), 1% glutamine (Invitrogen), 50 IU/mL penicillin and 50 IU/mL streptomycin (BioSource International, Camarillo, CA, USA) and under standard conditions (humidified atmosphere of 5% CO_2_ at 37°C). To obtain macrophages, THP-1 cells were seeded in six-well culture plates at 10^6^ cells/mL and stimulated with PMA (Sigma-Aldrich, St Louis, MO, USA) for 72 h at 100 nM as previously described [20]. In our experiments, approximately 10% of cells became differentiated and were selected for further analysis.

Wong-Kilbourne derivative (WKD) of Chang conjunctival epithelial cells (clone 1 to 5c-4l, American Type Culture Collection) were cultured under standard conditions (humidified atmosphere of 5% CO_2_ at 37°C) in Dulbecco's minimum essential medium (DMEM) supplemented with 10% fetal bovine serum (HyClone), 1% glutamine (Invitrogen), 50 IU/mL penicillin, and 50 IU/mL streptomycin (BioSource International) as previously described [21].

### Stimulations

As previously described, PMA-differentiated THP-1 macrophages were selected by keeping only the adherent cells to the plate [22]. The culture medium containing non-adherent cells was discarded and a new medium without PMA was added. Then, macrophages were exposed for 24 h to 32.8 nM (10^−5^%) benzalkonium chloride (BAK) (Sigma-Aldrich), 10 nM dinitrochlorobenzene (DNCB) (Sigma-Aldrich), 100 ng/mL lipopolysaccharide (LPS) (*Escherichia coli* serotype 0111: B4) (Sigma-Aldrich), 5 ng/mL tumor necrosis factor alpha (TNF-α) (PeproTech, Rocky Hill, NJ, USA) or phosphate buffered saline (PBS) as control. All reagents were diluted in PBS. Supernatants were harvested and stored at −20°C after 24 h of exposure.

### Dose response cytotoxicity evaluation in flow cytometry (FC)

The concentrations of stimulating factors (BAK, DNCB, LPS and TNF-α) were chosen after cell viability testing, using an Annexin V/7-aminoactinomycin D (7-AAD) double staining in flow cytometry (Cytomics FC 500-CXP, Beckman Coulter, Miami, FL, USA). The FC is equipped with an Argon laser at 488 nm. Annexin V binds only on phosphatidylserin, present on the external layer of plasmic membrane on the early stages of apoptosis [23]. 7-AAD is a fluorescent probe, which binds between nucleic acid of cells in necrosis or late apoptosis. PMA differentiated THP-1 cells were exposed to reagents with a gradient of concentration during 24 hours. BAK was used at 33 nM, 164 nM, 328 nM, and 1.6 µM (10^−5^%, 5.10^−5^%, 10^−4^% and 5.10^−4^%). DNCB was used at 10 nM, 25 nM, 50 nM and 100 nM. LPS was used at 10 ng/mL, 50 ng/mL, 100 ng/mL and 250 ng/mL. TNF-α was used at 1 ng/mL, 2 ng/mL, 5 ng/mL and 10 ng/mL. PBS was used for control. After exposure, cells were harvested with a cell scraper. Analysis was made with the help of the kit ANNEXIN V-FITC/7-AAD (Beckman Coulter) without PFA's fixation according to the manufacturer using FC. A biparametric histogram was used to determine annexin V and 7-AAD binding. Four populations were identified, those who stayed negative to both markers (viable cells), positive only to annexin V (early apoptosis), positive to both markers (late apoptosis) and positive only to 7-AAD (necrosis).

Concentrations just below toxicity levels were selected for each stimulating factor. The concentrations of BAK and DNCB used were similar to other studies on THP-1, which determined a low level of cell death [19], [24]. Concentrations of TNF-α and LPS were similar to other studies on THP-1 cells [25], [26].

### Expression of macrophage markers

To determine the cell phenotypes in each condition, the expression of cell markers was quantified in flow cytometry (FCM) (Beckman Coulter). THP-1 cells were harvested with a cell scraper and WKD cells were harvested after 10 min incubation with ethylenediaminetetraacetic acid (EDTA) at 0.5 mM (Sigma-Aldrich). Cells were then washed and fixed in 0.5% paraformaldehyde (PFA) in PBS (Alfa Aesar, Ward Hill, MA, USA) for 24 h at 4°C before immunostaining and FCM analysis. The monoclonal antibodies used were : fluorescein isothiocyanate (FITC)-conjugated CD11b, CD86, CD54 (Pharmingen, San Diego, CA, USA), phycoerythrin (PE)-conjugated CD11c (Pharmingen), PE-CD33 (BD Biosciences, San Jose, CA, USA) and an isotypic control antibody (mouse IgG1) (Pharmingen). For each condition 3×10^4^ cells were washed with PBS and suspended in 50 µl binding buffer containing 3 µL of fluorescent antibody for 30 min at room temperature. Cells were then washed in PBS and suspended in 200 µL buffer before FCM analysis.

The results are given as mean fluorescence intensity (MFI) ratios corresponding to the ratio between the MFI obtained for the antigen-specific antibody and the MFI obtained for the matched isotypic negative control. A positive expression corresponds to a ratio>1 and could also be observed in basal condition. Percentages of expression were not shown because the cell markers were expressed by more than 95% of the macrophages, thus only the intensities of expression are presented. To illustrate the phenotype of THP-1 differentiated cells, we chose to perform immunohistochemistry staining of CD11c. THP-1 differentiated cells were seeded on compartmented glass plates (Lab-Tek™, NUNC, Roskilde, Denmark) to visualize the expression of differentiation markers. We used 500 µL of cell suspension at 10^6^ cells/mL per compartment. Cells were fixed for 15 min in PBS-PFA at 4% and then washed three times with PBS. Half of the compartments were incubated with fluorescent (PE) mouse anti-CD11c (Pharmingen) diluted at 1/100 with PBS; the other half was incubated with a PBS solution at 1/100 of fluorescent (PE) isotypic control antibody anti-IgG1. After three more washes with PBS, the compartments were isolated and glass plates were mounted with a medium containing 4′,6-diamidino-2-phenylindole (DAPI) (Vectashield-DAPI; Lumigen Inc., Southfield, MI, USA). An epifluorescent microscope (DM 6000, Leica Microsystems GmbH, Wetzlar, Germany) showed staining of CD11c fluorescent antibodies.

### Phagocytosis

To study phagocytosis, differentiated and stimulated THP-1 cells were seeded in compartmented glass plates (Lab-Tek™ Nunc, Naperville, IL, USA) or six-well plates. Then they were incubated for 1 h with 100 µL of carboxylate-modified fluorescent polystyrene microspheres (580/605 nm wavelength, 0.2 µm; Invitrogen) at the concentration of 50 µg/mL. These spheres were earlier coated in standard culture conditions for 1 h in PBS containing 10% FBS for antigen recognition and phagocytosis. Cells cultured in glass plates were fixed with 0.5% PFA in PBS for 24 h after three 5-min washes with PBS, mounted on glass slides with Vectashield™-DAPI (Vector) and analyzed with an epifluorescent microscope. Cells cultured in six-well plates were harvested and fixed (0.5% PFA in PBS for 24 h) for FCM analysis. The cytometer equipped with Argon laser was used to count the microsphere-containing cells detecting the wavelength emission of the fluorescent microspheres. A monoparametric histogram displayed the percentage of cells containing microspheres.

### Cell migration

To evaluate the migration of macrophages, PMA-differentiated THP-1 cells were cultured on permeable membranes with 8.0- µm pores (inserts) (Falcon, BD Biosciences, Franklin Lakes, NJ, USA) in suspension (1 mL of each at 10^6^ cells/mL in RPMI medium) in six-well plates. WKD cells (at 60,000 cells/mL in DMEM medium) were also seeded in other six-well plates to 70–80% confluence and both cell lines were exposed to the same stimulants for 24 h. Then inserts with THP-1 cells were transferred into the six-well plates containing WKD conjunctival cells and both cells were cocultured for 4 h. One insert with THP-1 cells was transferred into a plate without WKD for control. Membranes were harvested, fixed with PBS-PFA at 4% for 15 min, gently washed with PBS and mounted on glass plates. Pictures of cells on the upper and lower side of each membrane were taken with an epifluorescent microscope. THP-1 cell migration was measured by comparing the ratio between the number of DAPI-stained nuclei on the upper and lower sides of each membrane. The nuclei counting was assessed with ImageJ® software (Wayne Rasband, National Institutes of Health, MD, USA).

### Cytokine production

Cytokines were analyzed in supernatants from differentiated and stimulated THP-1 cells. Supernatants were harvested 24 h after the last recovery of the medium. They were analyzed using a Proteome Profiler Array® (ARY005, R&D Systems, Minneapolis, MN, USA) that could identify 38 cytokines. Nitrocellulose membranes were spotted with various anticytokine antibodies. All experiments were done according to the manufacturer's instructions. One nitrocellulose membrane and 1 mL of supernatant were used for each condition. After incubation with the supernatant, membranes were stained with an electroluminescent agent (Amersham, GE Healthcare Limited, Buckinghamshire, UK) before revelation on X-ray film (Amersham, GE Healthcare Limited) according to the manufacturer's instructions. The pixel density of each spot was analyzed using ImageJ® software. The density of each spot was given as the mean from the ratio (%) versus control of three experiments.

### Statistical analysis

All experiments were conducted in triplicate. For multiple comparisons between different conditions, a Kruskal-Wallis nonparametric test with multiple impaired comparisons and Bonferroni correction was used. For simple comparisons between two conditions, a Student-unpaired *t*-test was used. *P*<0.05 was considered significant.

## Results

### THP-1 cells were differentiated into macrophages after PMA stimulation

**Figure 1:**
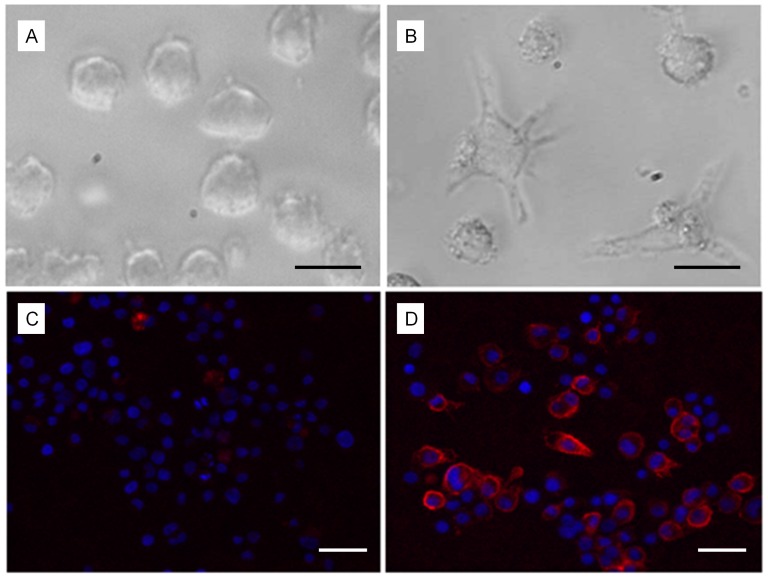
Light microscopy and fluorescence microscopy images of non-differentiated and differentiated THP-1 cells. Non-differentiated THP-1 cells (A) display a round shape and a nonadherent pattern while differentiated THP-1 cells (B) display a dendritic shape and an adherent pattern at light microscopy (×400), black scale bar : 10 µm. Fluorescence microscopy images (×200) showing CD11c expression by non-differentiated THP-1 cells (C) and differentiated THP-1 cells (D). Nuclei are stained in blue (DAPI) and CD11c is stained in red phycoerythrin (PE), white scale bar : 20 µm. A significantly increased CD11c expression was observed on differentiated THP-1 cells.

**Figure 2:**
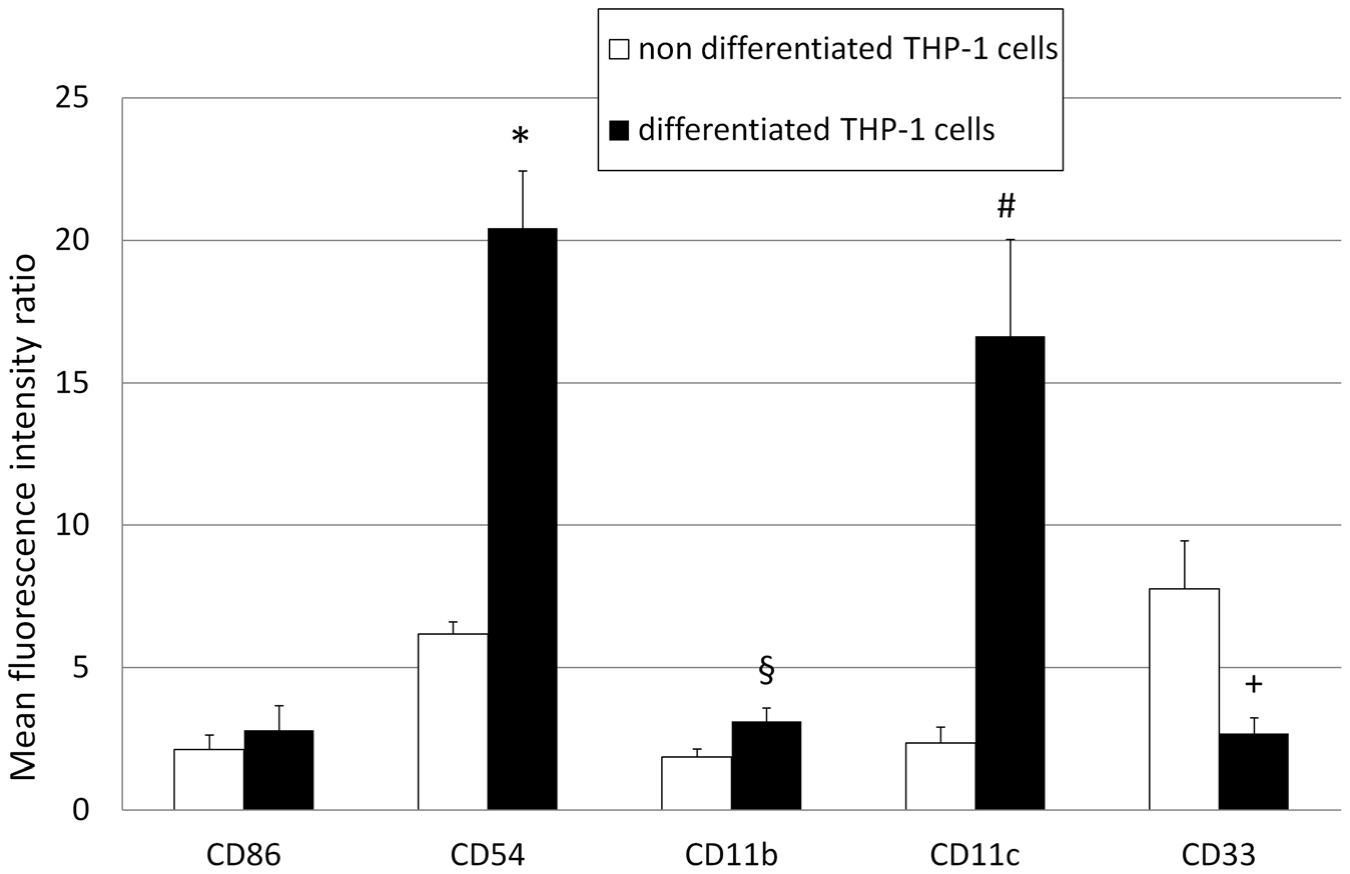
Quantification of fluorescence intensity by non-differentiated and differentiated THP-1 cells. Membrane expression of CD54 (*P* = 0.033, *), CD11b (*P* = 0.031, §) and CD11c (*P* = 0.002, #) was significantly increased by differentiated ▪ THP-1 cells compared to non-differentiated □ THP-1 cells. A significant decrease of CD33 expression (*P* = 0.049, +) were observed and no significant difference of CD86. Errors bars represent standard deviation.

After 72 h of exposure to PMA, THP-1 cell morphology was modified from a round and non adherent pattern to an adherent and dendritic pattern (Figure 1 A, B). Moreover, FCM quantification of differentiated THP-1 cells showed an increased expression of CD11b (*P* = 0.031), CD11c (*P* = 0.033) and CD54 (*P* = 0.002), and a decreased expression of CD33 (*P* = 0.049) as compared to undifferentiated THP-1 cells (Figure 2). The differentiated THP-1 phenotype was also illustrated by the increased expression of CD11c in immunochemistry (Figure 1C, D). No significant difference was found in the expression of CD86.

### BAK and DNCB had a dose dependent cytotoxicity on PMA-differentiated THP-1 cells

Total cytotoxity expressed by PMA differentiated THP-1 cells exposed during 24 hours to BAK at 33 nM was 39.3±4% and to DNCB at 10 nM was 29.5±1.8% (Table 1). Those percentages were not significantly different compared to control (PBS), 33±2.7% (*P* = 0.075 for BAK and *P* = 0.112 for DNCB). The other concentrations of BAK and DNCB tested increased total cytotoxicity significantly (*P* = 0.019, 0.009 and 0.006 for BAK 164 nM, 328 nM and 1.6 µM respectively and *P* = 0.017, 0.011 and 0.005 for DNCB 25 nM, 50 nM and 100 nM respectively) compared to PBS. According to these conclusions we decided to choose 10^−5^% for BAK and 10 nM for DNCB in further investigations. LPS and TNF-α had no cytotoxic effects on PMA-differentiated THP-1 cells compared to PBS (*P* = 0.377 for LPS at 100 ng/mL and *P* = 0.092 for TNF-α at 5 ng/mL) (Table 1).

**Table 1:** Percentage of cytotoxicity of PMA differentiated THP-1 exposed to reagents.

Reagent	Concentration	early apoptosis (%)	late apoptosis (%)	necrosis (%)	total (%)
**PBS**	1x	8.3±3.5	15.3±1.5	9.3±2.5	33±2.7
**BAK**	33 nM	9.3±2.6	19.6±4.5	10.3±2.51	39.3±4
	164 nM	10±3.5	**25.6±0.6**	11.3±2.3	**47±3.1**
	328 nM	11.6±3.1	**31±4.7**	10±1	**52.6±8.2**
	1.64 µM	12.5±2.82	**36±4.2**	**13.5±0.7**	**62±9.1**
**DNCB**	10 nM	10.1±1.9	11±3.6	8.4±2.6	29.5±1.8
	25 nM	15.6±8.5	15.6±4.7	**12.6±2.4**	**44±4.7**
	50 nM	13.3±5.4	**32.7±6.5**	**16.8±3.2**	**62.8±6.1**
	100 nM	10±7.54	**34.6±5.5**	**25.5±3.8**	**70.2±8.5**
**LPS**	10 ng/mL	9.6±0.5	13.6±1.1	9±1.7	32.3±2.1
	50 ng/mL	10.6±0.6	15±2.65	7.6±2.5	33.3±4.1
	100 ng/mL	9.3±1.1	15.3±3.5	8.6±2.1	33.3±4.5
	250 ng/mL	8.5±2.1	15.5±0.7	9±1.4	33±1.4
**TNF-α**	1 ng/mL	9.6±2.1	17±1.73	17±3.6	36.6±3.2
	2 ng/mL	9±2.65	18±2.65	9.6±3.5	36.6±1.5
	5 ng/mL	11.3±2.5	17.6±0.6	7.6±3.8	36.6±2.1
	10 ng/mL	9.6±2.3	17±1	9.6±3.7	36.3±2.5

Cell viability of PMA differentiated THP-1 was measured using an Annexin V/7-aminoactinomycin D (7-AAD) double staining in flow cytometry. We tested the 24 hours exposure to benzalkonium chloride (BAK), dinitrochlorobenzen (DNCB), lipopolysaccharide (LPS), tumor necrosis factor alpha (TNF-α) and phosphate buffered saline (PBS) for control. For each reagent, a panel of 4 different concentrations was tested. Grades of toxicity were separated in early apoptosis, late apoptosis, necrosis and total cytotoxicity. Results are expressed in percentage ± standard deviation (SD). Results with statistical differences (*P*<0.05) compared to PBS are presented in bold.

### The macrophage phenotype was modified after exposure to BAK, PMA, DNCB, LPS and TNF-α

**Figure 3:**
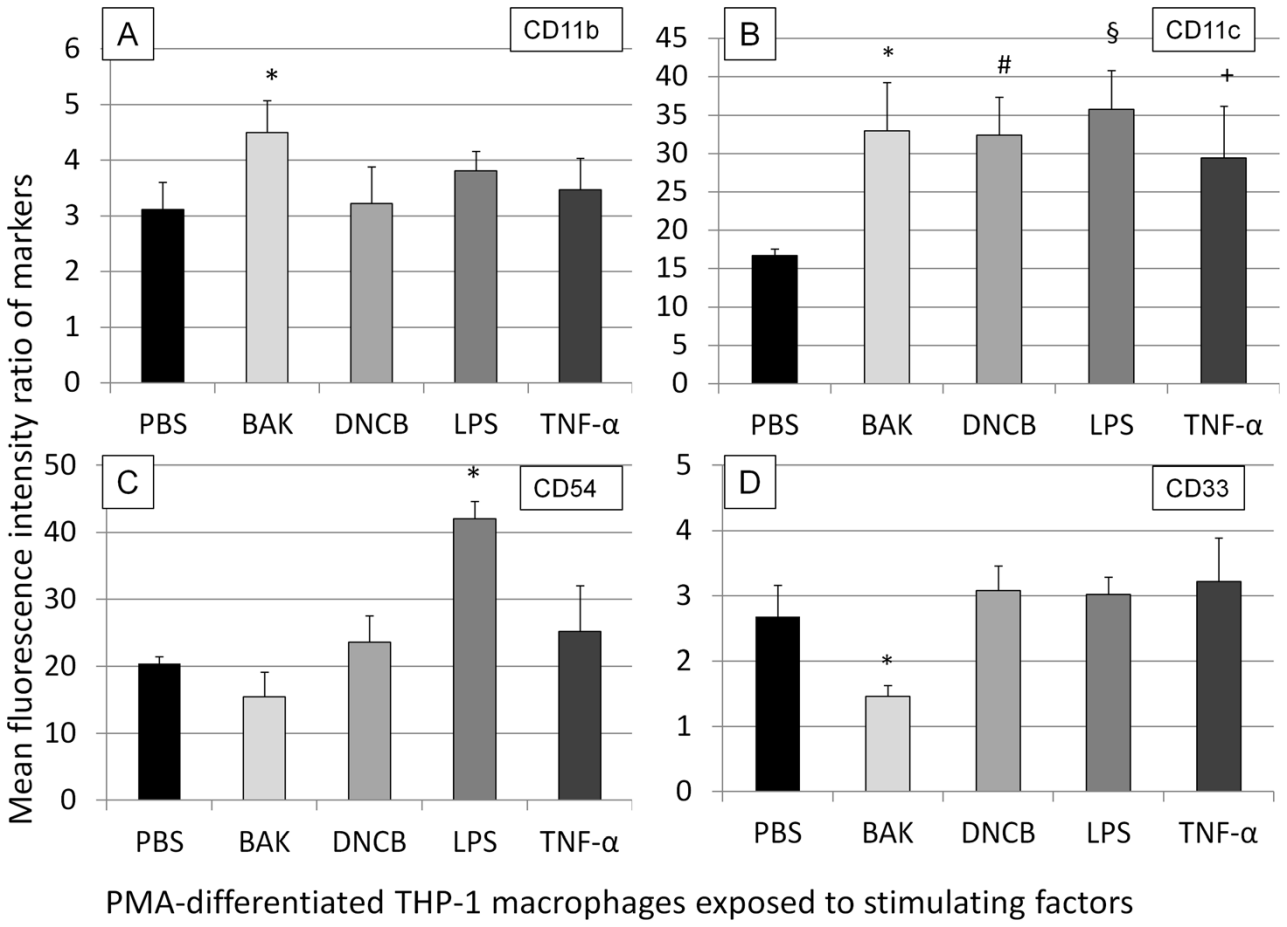
Flow cytometry analysis of phenotypic changes of macrophages after various stimulatory substances. A: expression of CD11b : the expression of CD11b increased after benzalkonium chloride (BAK) stimulation (*P* = 0.033, *) as compared to phosphate buffered saline (PBS). B: expression of CD11c : the expression of CD11c increased with BAK (*P* = 0.005, *), DNCB (*P* = 0.004, #), lipopolysaccharide (LPS) (*P* = 0.035, §) or tumor necrosis factor alpha (TNF-α) (*P* = 0.033, +) as compared to PBS. C: expression of CD54 : the expression increased under LPS stimulation (*P* = 0.007, *) as compared to PBS. D: expression of CD33 : a decreased expression of CD33 was observed when exposed to BAK (*P* = 0.003, *) as compared to PBS. Errors bars represent standard deviation.

The membrane expression of CD11b by differentiated macrophages was selectively increased after exposure to BAK (*P* = 0.033) as compared to PBS and other tested stimulations (Figure 3, A). In contrast, expression of CD11c was also increased after incubation with BAK (*P* = 0.005), DNCB (*P* = 0.004), LPS (*P* = 0.035) and TNF-α (*P* = 0.033) as compared to PBS (Figure 3, B). The membrane expression of CD54 was selectively increased after LPS stimulation (*P* = 0.007) as compared to PBS (Figure 3, C). Furthermore, only when exposed to BAK, CD33 expression was reduced (*P* = 0.003) as compared to PBS (Figure 3, D).

### Macrophages exposed to BAK, LPS and TNF-α showed increased phagocytosis

**Figure 4:**
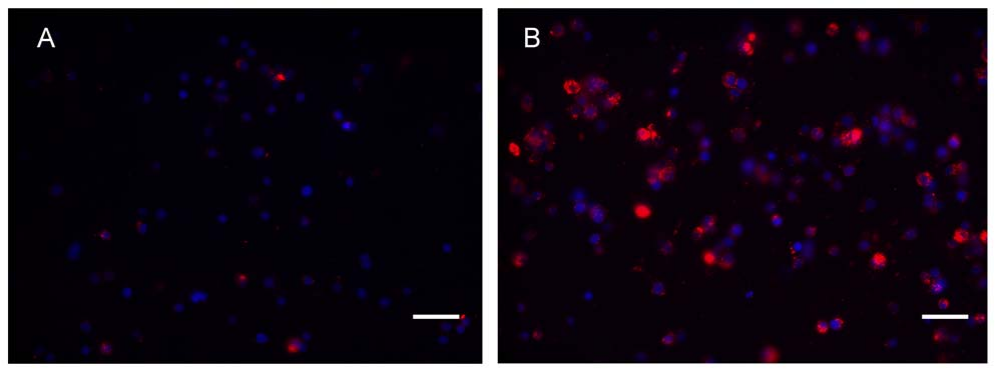
Fluorescence microscopy images of carboxylate microspheres in non-differentiated THP-1 cells and in macrophages. An increased carboxylate microspheres (red) concentration is observed in macrophages (B) than in non-differentiated THP-1 cells (A). Nuclei are stained in blue (DAPI). Magnification used was ×400. Bar : 20 µm.

**Figure 5:**
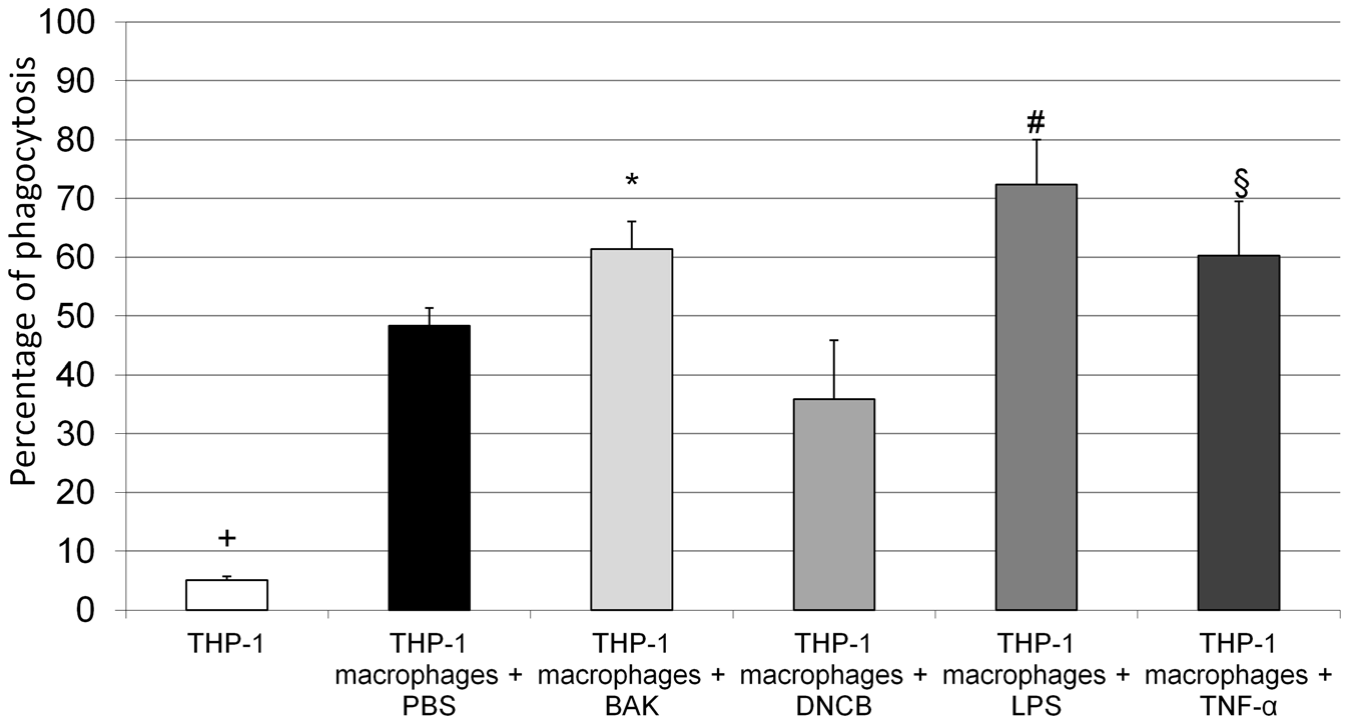
Quantification of carboxylate-modified fluorescent microspheres in non-differentiated THP-1 cells and macrophages. Macrophages had a higher level of phagocytosis (48.3±3%) (*P*<0.0001, +) as compared to non-differentiated THP-1 cells (5.1±0.6%). Increased phagocytosis was observed after 24-h exposure to benzalkonium chloride (BAK) (*P* = 0.015, *), lipopolysaccharide (LPS) (*P*<0.0001, #) or tumor necrosis factor alpha (TNF-α) (*P* = 0.03, §) as compared to macrophages exposed to phosphate buffered saline (PBS). Errors bars represent standard deviation.

By means of fluorescence microscopy, a higher number of fluorescent microspheres were observed in macrophages as compared to non-differentiated THP-1 cells (Figure 4). Quantification with FCM confirmed that the percentage of cells containing fluorescent microspheres was significantly higher (*P* = 0.005) in macrophages (48.3±3%) than in non-differentiated THP-1 cells (5.1±0.6%). Furthermore, phagocytosis was significantly increased when macrophages were exposed to BAK (61.4±4.7%, *P* = 0.015), LPS (72.4±7.6%, *P*<0.0001) or TNF-α (60.2±9.3%, *P* = 0.03) as compared to macrophages exposed to PBS (48.3±3%) (Figure 5).

### Macrophages showed increased migration under the influence of BAK and TNF-α

**Figure 6:**
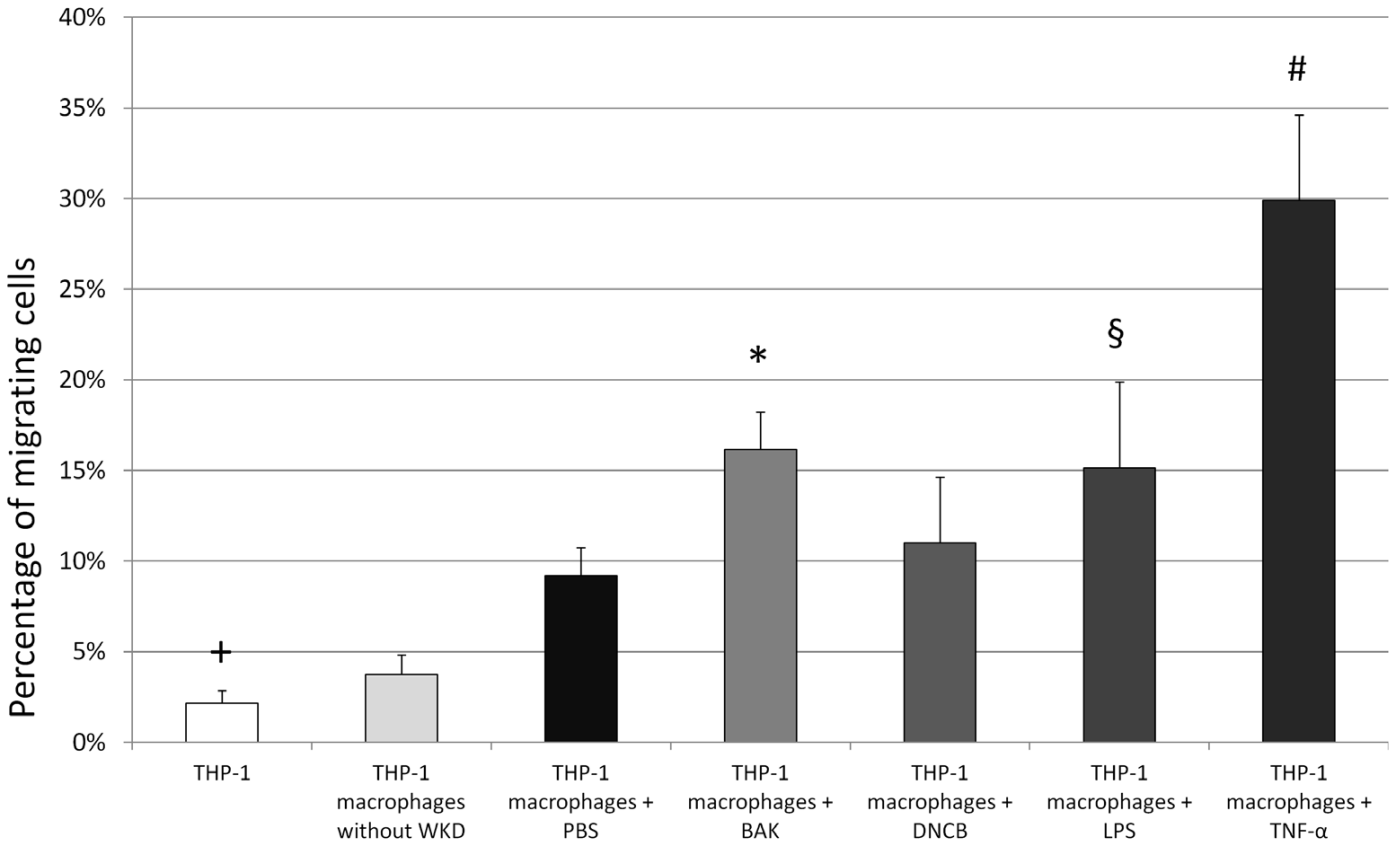
Evaluation of migration rates of non-differentiated THP-1 cells and PMA differentiated macrophages. The migration rate was higher when macrophages were exposed to benzalkonium chloride (BAK) (16.2±2.1%, *P* = 0.001,*), lipopolysaccharide (LPS) (15.1%±4.7%, *P* = 0.004, §) or tumor necrosis factor alpha (TNF-α) (29.9±4.7%, *P*<0.0001, #) as compared to macrophages exposed to phosphate buffered saline (PBS). Migration was significantly higher when macrophages were exposed to TNF-α as compared to BAK (*P* = 0.004). Migration was measured in coculture with conjunctival cells except for column 2. Errors bars represent standard deviation.

We found that macrophages had a significantly increased rate of migration (9.2±1.5%, *P* = 0.004) when cocultured in presence of conjunctival cells unlike non-differentiated THP-1 cells (2.1±0.7%) and PMA-differentiated THP-1 cells cultured without conjunctival cells (3.7±1.1%). Migration rates were increased when macrophages were exposed to BAK (16.2±2.1%, *P* = 0.001), LPS (15.1%±4.7%, *P* = 0.004) or TNF-α (29.9±4.7%, *P*<0.0001) as compared to macrophages exposed to PBS. Migration was significantly greater when macrophages were exposed to TNF-α (Figure 6).

### Cytokine expression by macrophages was modified after exposure to stimulating factors

Supernatants from non-differentiated THP-1 cells contained high levels of CCL2/MCP-1 and MIF (over 50% of the positive control) and low detectable levels of IL-1ra, IL-8, PAI-1 and CCL5 (approximately 10% of the positive control) (Table 2). In addition to the few cytokines found in non-differentiated THP-1 cells, supernatants of macrophages contained high levels of C5a, CXCL10, IL-1ra, CCL3, CCL5 and MIF (over 65% of the positive control) and detectable levels of CCL1, soluble CD54, GM-CSF and TNF-α (between 15 and 35% of the positive control). After exposure to the different stimulating factors, supernatants from macrophages contained more various cytokines and showed higher levels of expression. Macrophages exposed to BAK and TNF-α induced CCL1, soluble CD54, IL-1β, CCL4 and TNF-α at high levels (over 65% of the positive control) and CXCL11 at a low level (19% and 17% of the positive control, with BAK and TNF-α, respectively). Exposure to DNCB and LPS showed a substantial release of CXCL1 and IL-16. Moreover, supernatants from cells exposed to LPS (50 ng/mL) contained high levels of G-CSF, IL-1α, IL-6 and IL-10. Exposure to LPS also showed detectable levels of IFN-γ, IL-23, CXCL12 and sTREM-1. The results are presented in Table 2.

**Table 2:** Quantification of cytokines in supernatants from non differentiated and PMA-differentiated THP-1 cells.

THP-1	Non differentiated	PMA-differentiated	PMA-differentiated	PMA-differentiated	PMA-differentiated	PMA-differentiated
Cytokines	PBS	PBS	BAK	DNCB	LPS	TNF-α
**C5a**	0	81±9	74±3	91±1	89±4	64±11
**CD40L**	0	1±2	0	18±11	13±8	0
**GCSF**	0	0	0	8±3	**63±13**	0
**GMCSF**	0	19±6	17±3	19±7	**87±12**	9±4
**CXCL1**	0	8±2	13±2	**68±3**	**87±8**	9±2
**CCL1**	0	31±9	**73±10**	**94±8**	**90±8**	74±7
**CD54**	0	23±4	**72±9**	**93±5**	93±4	69±6
**IFN-y**	0	0	0	12±8	12±7	0
**IL-1 α**	0	0	0	13±9	**84±5**	0
**IL-1 β**	0	5±1	**74±6**	**89±5**	**86±4**	**71±5**
**IL-1ra**	5±1	90±12	74±7	88±2	86±2	75±5
**IL-2**	0	0	0	11±7	7±2	0
**IL-4**	0	0	0	3±1	3±1	0
**IL-5**	0	0	0	3±1	3±1	0
**IL-6**	0	8±2	1	8±6	**93±7**	7±3
**IL-8**	8±2	84±11	72±4	89±4	82±5	60±4
**IL-10**	0	0	0	**17±6**	**43±13**	0
**IL-12 p70**	0	0	0	**13±2**	**19±5**	0
**IL-13**	0	0	0	**15±4**	**11±1**	0
**IL-16**	0	2	9±2	**60±2**	**65±5**	3±1
**IL-17**	0	0	0	4±1	4±1	0
**IL-25**	0	0	0	7±2	4±1	0
**IL-23**	0	0	0	8±1	**15±4**	0
**IL-27**	0	0	0	5±1	6±1	0
**IL-32α**	0	0	0	4±1	5±2	0
**CXCL10**	0	49±4	**75±6**	**91±5**	**86±2**	**76±3**
**CXCL11**	0	0	**19±7**	**14±1**	**21±3**	**17±2**
**MCP-1**	88±6	84±11	74±5	91±6	87±5	70±5
**MIF**	51±10	76±7	75±4	85±6	90±5	**74±6**
**CCL3**	0	76±13	75±6	89±7	89±5	74±7
**CCL4**	0	8±4	**75±13**	**90±4**	**86±4**	74±6
**PAI-1**	11±2	79±9	74±9	85±6	81±7	73±8
**CCL5**	6±1	94±6	75±7	86±5	78±1	76±4
**CXCL12**	0	0	0	**18±4**	**18±5**	0
**TNF-α**	0	15±3	**70±7**	**88±8**	**81±5**	**84±4**
**sTREM-1**	0	0	0	**25±5**	**25±5**	0

Quantification of cytokines in supernatants from undifferentiated THP-1 cells and macrophages (differentiated THP-1 cells) stimulated with benzalkonium chloride (BAK), dinitrochlorobenzene (DNCB), lipopolysaccharide (LPS) or tumor necrosis factor alpha (TNF-α), and phosphate buffered saline (PBS) as a control. Results are expressed as a percentage of expression compared to the negative control ± standard deviation (SD). SD was not shown if <1. Results with statistical differences (*P*<0.05) compared to macrophages exposed to PBS are presented in bold.

## Discussion

In this *in vitro* study, THP-1 cells were differentiated into macrophages, according to a well-known procedure [20], [22]. THP-1 cells were previously shown to be a widely used cell line to investigate function and regulation of macrophages *in vitro* [27], [28]. Regarding the association of morphological and functional markers showed by differentiated THP-1 cells, these cells are commonly used as antigen-presenting cells *in vitro* [29]. In the present study, we selected THP-1 differentiated cells adherent to the cell plate as previously described [30]. These cells had a dendritic morphology and adhesion abilities, characteristics shared by dendritic cells and macrophages [2]. In our study, the morphological changes were associated with the expression of CD11b, a macrophage marker and CD11c a dendritic cell marker [2] that were both overexpressed by differentiated THP-1 cells. Furthermore, a decreased expression of CD33, a myeloid cell marker that decreases with cell differentiation, confirmed the differentiated phenotype of these cells [31]. Differentiated THP-1 cells also demonstrated migration and phagocytosis abilities common to antigen-presenting cells *in vitro*.

BAK is widely used in eye drops as a preservative, with known toxic and pro-inflammatory effects on ocular surface tissues [7], [32]. When exposed to BAK at a concentration of 329 nM (10^−4^%), conjunctival and corneal epithelial cell growth was reduced and cells became apoptotic at higher concentrations [9], [21]. BAK is also responsible for the production of inflammatory mediators and proinflammatory cytokines by inflammatory skin cells with a direct relationship between clinical signs of irritation and *in vitro* release of the proinflammatory mediators [33]. In the present *in vitro* study, a low concentration of BAK (33 nM) was used in order to be below the direct cytotoxic effects on macrophages and to better reproduce the *in vivo* effects of BAK on ocular surface tissues. Thus, we showed that low concentrations of BAK stimulated macrophages regarding migration, cytokine production, phagocytosis and expression of cell markers.

Using the similar THP-1 cell line, Hirota *et al.* observed that 330 nM (10^−4^%) BAK did not induce CCL4 (MIP-1β) and CD86 production by undifferentiated THP-1 cells *in vitro* [18]. More interestingly, it was reported that 1 µM (3.2×10^−4^%) BAK was able to induce a 50% inhibitory effect on THP-1 cell proliferation [24]. This concentration of BAK decreased CCL4 production by undifferentiated THP-1 cells, whereas lower concentrations (100 nM and 10 nM, respectively, 3.2×10^−5^% and 3.2×10^−6^%) had no effect on CCL4 production. Although, we similarly observed that a low concentration of BAK (33 nM or 10^−5^%) did not induce CCL4 production by THP-1 cells, this concentration of BAK could induce cytokines production by macrophages (THP-1 differentiated cells). It has been previously shown that BAK induced the release of inflammatory cytokines (IL-1β, CXCL1, CCL3, CCL5) and IL-1β, CXCL8/IL-8 and CCL2/MCP-1 by vaginal parenchyma [34], [35]. On ear tissues, application of BAK has been shown to release inflammatory cytokines such as TNF-α, IL-1β and MIP-1α and MIP-1β [36]. On ocular surface cells, BAK induced the production of IL-1 and TNF-α by conjunctival and corneal cells [37]. In the present study, supernatants from macrophages stimulated with BAK demonstrated high levels of ICAM-1/CD54, CXCL8/IL-8, CCL4, IL-1β and TNF-α. Interestingly, ICAM-1 and IL-8 were also found on impression cytology from patients treated with eye drops containing BAK [8]. Consistently, BAK induced cytokine production by macrophages similar to those found in other tissues exposed to BAK [34]–[37]. These new results emphasize the stimulating effects of BAK on macrophages *in vitro*.

To compare the effects of BAK on macrophages, other stimulating factors mimicking stimulations that may occur in ocular surface diseases were used: DNCB, TNF-α and LPS. DNCB, a benzene-derived allergen, is responsible for contact dermatitis [38]. It has a stimulating role for antigen-presenting cells, increasing expression of cell markers such as HLA-DR, CD86, CD40 and CD54, T-lymphocyte proliferation, TNF-α production, and Cys-Cys chemokine receptor 7 (CCR7) expression [39]. DNCB is considered as an allergen *in vivo*, regarding its pro-inflammatory action on macrophages [38], [39]. Most notably with macrophages, we observed that DNCB induced an overexpression of CD11c and the release of pro-inflammatory cytokines (CXCL1, IL-16, CCL1, soluble CD54, IL-1β, CCL4 and TNF-α), but DNCB had no effect on migration and phagocytosis. Lipopolysaccharides are expressed on the external layer of Gram-negative bacteria. Their pro-inflammatory effects on conjunctival cells and antigen-presenting cells have been widely demonstrated [40], [41]. TNF-α is a mediator of inflammation produced by various cell types, which acts by inducing cytokine production, adhesion and costimulation molecules by inflammatory cells [42], [43]. LPS and TNFα are both well-known stimulating factors for macrophage functions such as migration, phagocytosis and cytokine production [40]–[43]. Interestingly, in the present study, BAK, LPS and TNF-α stimulations of CD11b and CD11c expression by macrophages were not different. Similarly, considering phagocytosis, no difference was observed between the stimulating effects of BAK, LPS and TNF-α. LPS was the most stimulating factor of cytokine release by macrophages and also increased CD54 expression, which was not modified by BAK, TNF-α or DNCB. For macrophage migration, TNF-α had the highest stimulating effect as compared to BAK and LPS. Although variations may exist in the type of macrophage properties evaluated, low concentrations of BAK may act on ocular surface macrophages as an activating factor comparable to TNF-α and LPS.

BAK has been considered as a cytotoxic agent regarding its effects on conjunctival cells [9] but has not been considered yet as a direct stimulating factor of inflammatory cells [7]. Low concentrations of BAK, as can be observed in patients treated over the long term with preserved antiglaucoma medications, could thus be directly responsible for recruiting macrophages and increasing their migratory behavior. Interestingly, in our study, macrophages exposed to BAK increased migration when cocultured with WKD conjunctival epithelial cells. Even if the role of BAK was not direct, it may influence migration of macrophages, as it has been previously demonstrated for TNF-α that was shown to regulate the migration of corneal dendritic cells [43]. The migration effect of BAK could be related to the TNF-α release induced by BAK in the present study, as we have a TNF-α secretion induced by BAK [18], [34]. Little is known about the effect of BAK on macrophage phagocytosis function. Conjunctival cells exposed to BAK contain autophagosomes and autolysosomes [44], leading to phagocytosis by other cells. *In vitro*, we observed that macrophage phagocytosis was enhanced by BAK exposure. The overexpression of CD11b and CD11c, facilitating the phagocytosis of complement-opsonized particles [45], might explain this effect of BAK on macrophages. Thus, in ocular surface tissues, BAK could activate phagocytosis by inducing apoptosis/autophagy in epithelial cells and also directly stimulating macrophages.

These results suggest new mechanisms involving macrophages that could be in part responsible for the inflammatory reactions observed when ocular surface tissues are exposed to BAK. It has been previously shown that BAK was able to induce an infiltration of macrophages in the conjunctiva and the expression of inflammatory and fibroblastic markers in patients treated for glaucoma or ocular hypertension [12], [13], [15]. In rabbit conjunctiva model, antiglaucoma eye drops induced significant inflammatory cell infiltration in conjunctiva-associated lymphoid tissue related to the concentration of BAK [46]. In the present *in vitro* study, we used a concentration of BAK (33 nM) lower than the concentrations found in antiglaucoma eye drops (13 µM to 82 µM) [7]. However, in patients using preserved antiglaucoma eyedrops, because of the tear film dilution and the deeper location of macrophages as compared to superficial conjunctival epithelial cells, ocular surface macrophages and dendritic cells may also be exposed to lower concentrations of BAK. The pro-inflammatory effects induced by preserved eye drops and BAK in particular are responsible for ocular surface symptoms that are a significant barrier to compliance in glaucoma treatment. Moreover, macrophage infiltration of the conjunctiva was already pointed out as being responsible, at least in part, for the abnormally excessive scarring process observed after glaucoma filtering surgery [15], [47]. The present results emphasize the role of BAK in these conjunctival tissue changes. Considering the stimulating role of BAK on macrophages, BAK should be avoided before filtering surgery, at least in the preoperative period.

Identifying all cellular populations and their respective role is necessary to establish an accurate tissue model. The present study has shown that even if macrophages are not among the first cells exposed to BAK because they are located deep in the conjunctiva stroma, they undergo modifications in response to stimulation at even extremely low BAK concentrations. Interactions between these cells and other cellular populations throughout the ocular surface and in other locations in the conjunctiva and the lymph nodes need to be elucidated to better understand the overall effects of BAK on the ocular surface.

## References

[1] Research in dry eye: report of the Research Subcommittee of the International Dry Eye WorkShop. Ocul Surf 5: 179–93. 17508121 10.1016/s1542-0124(12)70086-1

[2] Forrester JV, Xu H, Kuffová L, Dick AD, McMenamin PG (2010) Dendritic cell physiology and function in the eye. Immunol Rev 234: 282–304. 20193026 10.1111/j.0105-2896.2009.00873.x

[3] Ishida W, Fukuda K, Kajisako M, Takahashi A, Sumi T, et al (2010) Conjunctival macrophages act as antigen-presenting cells in the conjunctiva during the development of experimental allergic conjunctivitis. Mol Vis 16: 1280–1285. 20664704 PMC2904043

[4] Pong JC, Chu CY, Li WY, Tang LY, Li L, et al (2011) Association of hemopexin in tear film and conjunctival macrophages with vernal keratoconjunctivitis. Arch Ophthalmol 129: 453–461. 21482871 10.1001/archophthalmol.2011.41

[5] Abu El-Asrar AM, Al-Kharashi SA, Missotten L, Geboes K (2006) Expression of growth factors in the conjunctiva from patients with active trachoma. Eye (Lond) 20: 362–369. 15818386 10.1038/sj.eye.6701884

[6] Tesavibul N, Dorfman D, Sangwan VS, Christen W, Panayotis Z, et al (1998) Costimulatory molecules in ocular cicatricial pemphigoid. Invest Ophthalmol Vis Sci 39: 982–988. 9579477

[7] Baudouin C, Labbé A, Liang H, Pauly A, Brignole-Baudouin F (2010) Preservatives in eyedrops: the good, the bad and the ugly. Prog Retin Eye Res 29: 312–334. 20302969 10.1016/j.preteyeres.2010.03.001

[8] Baudouin C, Liang H, Hamard P, Riancho L, Creuzot-Garcher C, et al (2008) The ocular surface of glaucoma patients treated over the long term expresses inflammatory markers related to both T-helper 1 and T-helper 2 pathways. Ophthalmology 115: 109–115. 17532048 10.1016/j.ophtha.2007.01.036

[9] Ammar DA, Noecker RJ, Kahook MY (2011) Effects of benzalkonium chloride- and polyquad-preserved combination glaucoma medications on cultured human ocular surface cells. Adv Ther 28: 501–510. 21603985 10.1007/s12325-011-0029-x

[10] Leung EW, Medeiros FA, Weinreb RN (2008) Prevalence of ocular surface disease in glaucoma patients. J Glaucoma 17: 350–355. 18703943 10.1097/IJG.0b013e31815c5f4f

[11] Malvitte L, Montange T, Vejux A, Baudouin C, Bron AM, et al (2007) Measurement of inflammatory cytokines by multicytokine assay in tears of patients with glaucoma topically treated with chronic drugs. Br J Ophthalmol 91: 29–32. 16943231 10.1136/bjo.2006.101485 PMC1857565

[12] Broadway DC, Grierson I, O′Brien C, Hitchings RA (1994) Adverse effects of topical antiglaucoma medication. I. The conjunctival cell profile. Arch Ophthalmol 112: 1437–1445. 7980133 10.1001/archopht.1994.01090230051020

[13] Sherwood MB, Grierson I, Millar L, Hitchings RA (1989) Long-term morphologic effects of antiglaucoma drugs on the conjunctiva and Tenon's capsule in glaucomatous patients. Ophthalmology 96: 327–335. 2710524 10.1016/s0161-6420(89)32888-0

[14] Broadway DC, Grierson I, O′Brien C, Hitchings RA (1994) Adverse effects of topical antiglaucoma medication. II. The outcome of filtration surgery. Arch Ophthalmol 112: 1446–1454. 7980134 10.1001/archopht.1994.01090230060021

[15] Baudouin C, Pisella PJ, Fillacier K, Goldschild M, Becquet F, et al (1999) Ocular surface inflammatory changes induced by topical antiglaucoma drugs: human and animal studies. Ophthalmology 106: 556–563. 10080214 10.1016/S0161-6420(99)90116-1

[16] Hamanaka T, Kasahara K, Takemura T (2011) Histopathology of the trabecular meshwork and Schlemm's canal in primary angle-closure glaucoma. Invest Ophthalmol Vis Sci 52: 8849–61. 21960557 10.1167/iovs.11-7591

[17] Brignole-Baudouin F, Desbenoit N, Hamm G, Liang H, Both JP, et al (2012) A new safety concern for glaucoma treatment demonstrated by mass spectrometry imaging of benzalkonium chloride distribution in the eye, an experimental study in rabbits. PLoS One 7: e50180. 23209668 10.1371/journal.pone.0050180 PMC3507684

[18] Hirota M, Moro O (2006) MIP-1beta, a novel biomarker for in vitro sensitization test using human monocytic cell line. Toxicol In Vitro 20: 736–742. 16314067 10.1016/j.tiv.2005.10.013

[19] Lim YM, Moon SJ, An SS, Lee SJ, Kim SY, et al (2008) Suitability of macrophage inflammatory protein-1beta production by THP-1 cells in differentiating skin sensitizers from irritant chemicals. Contact Dermatitis 58: 193–198. 18353026 10.1111/j.1600-0536.2007.01311.x

[20] Auwerx J (1991) The human leukemia cell line, THP-1: a multifaceted model for the study of monocyte-macrophage differentiation. Experientia 47: 22–31. 1999239 10.1007/BF02041244

[21] Brasnu E, Brignole-Baudouin F, Riancho L, Warnet JM, Baudouin C (2008) Comparative study on the cytotoxic effects of benzalkonium chloride on the Wong-Kilbourne derivative of Chang conjunctival and IOBA-NHC cell lines. Mol Vis 14: 394–402. 18334956 PMC2268853

[22] Kurosaka K, Watanabe N, Kobayashi Y (1998) Production of proinflammatory cytokines by phorbol myristate acetate-treated THP-1 cells and monocyte-derived macrophages after phagocytosis of apoptotic CTLL-2 cells. J Immunol 161: 6245–9. 9834112

[23] Vermes I, Haanen C, Steffens-Nakken H, Reutelingsperger C (1995) A novel assay for apoptosis: flow cytometric detection of phosphatidylserine expression on early apoptotic cells using fluorescein labelled annexin-V. J Immunol Methods 184: 39–51. 7622868 10.1016/0022-1759(95)00072-i

[24] Byamba D, Kim TG, Kim DH, Je JH, Lee MG (2010) The Roles of Reactive Oxygen Species Produced by Contact Allergens and Irritants in Monocyte-derived Dendritic Cells. Ann Dermatol 22: 269–78. 20711262 10.5021/ad.2010.22.3.269 PMC2917679

[25] Holter W, Goldman CK, Casabo L, Nelson DL, Greene WC, et al (1987) Expression of functional IL 2 receptors by lipopolysaccharide and interferon-gamma stimulated human monocytes. J Immunol 138: 2917–22. 3106493

[26] Miyazawa M, Ito Y, Kosaka N, Nukada Y, Sakaguchi H, et al (2008) Role of TNF-alpha and extracellular ATP in THP-1 cell activation following allergen exposure. J Toxicol Sci 33: 71–83. 18303186 10.2131/jts.33.71

[27] Tsuchiya S, Yamabe M, Yamaguchi Y, Kobayashi Y, Konno T, et al (1980) Establishment and characterization of a human acute monocytic leukemia cell line (THP-1). Int J Cancer 26: 171–176. 6970727 10.1002/ijc.2910260208

[28] Daigneault M, Preston JA, Marriott HM, Whyte MK, Dockrell DH (2010) The Identification of Markers of Macrophage Differentiation in PMA-Stimulated THP-1 Cells and Monocyte-Derived Macrophages. PLoS One 5: e8668. 20084270 10.1371/journal.pone.0008668 PMC2800192

[29] Estrella JL, Kan-Sutton C, Gong X, Rajagopalan M, Lewis DE, et al (2011) A Novel in vitro Human Macrophage Model to Study the Persistence of Mycobacterium tuberculosis Using Vitamin D(3) and Retinoic Acid Activated THP-1 Macrophages. Front Microbiol 2: 67. 21747789 10.3389/fmicb.2011.00067 PMC3128978

[30] Tominaga T, Suzuki M, Saeki H, Matsuno S, Tachibana T, et al (1998) Establishment of an activated macrophage cell line, A-THP-1, and its properties. Tohoku J Exp Med 186: 99–119. 10223614 10.1620/tjem.186.99

[31] Garnache-Ottou F, Chaperot L, Biichle S, Ferrand C, Remy-Martin JP, et al (2005) Expression of the myeloid-associated marker CD33 is not an exclusive factor for leukemic plasmacytoid dendritic cells. Blood 105: 1256–64. 15388576 10.1182/blood-2004-06-2416

[32] Nuzzi R, Vercelli A, Finazzo C, Cracco C (1995) Conjunctiva and subconjunctival tissue in primary open-angle glaucoma after long-term topical treatment: an immunohistochemical and ultrastructural study. Graefes Arch Clin Exp Ophthalmol 233: 154–162. 7758983 10.1007/BF00166608

[33] Van de Sandt JJM, Maas WJM, Doornink PC, Rutten AAJJL (1995) Release of arachidonic and linoleic acid metabolites in skin organ cultures as characteristics of in vitro skin irritancy. Fundam Appl Toxicol 25: 20–28. 7601324 10.1006/faat.1995.1036

[34] Cone RA, Hoen T, Wong X, Abusuwwa R, Anderson DJ, et al (2006) Vaginal microbicides: detecting toxicities in vivo that paradoxically increase pathogen transmission. BMC Infect Dis 6: 90. 16740164 10.1186/1471-2334-6-90 PMC1523343

[35] Alt C, Harrison T, Dousman L, Fujita N, Shew K, et al (2009) Increased CCL2 expression and macrophage/monocyte migration during microbicide-induced vaginal irritation. Curr HIV Res 7: 639–649. 19929801 10.2174/157016209789973682 PMC2892884

[36] Roebrock K, Wolf M, Bovens S, Lehr M, Sunderkötter C (2012) Inhibition of benzalkonium chloride-induced skin inflammation in mice by an indol-1-ylpropan-2-one inhibitor of cytosolic phospholipase A2 α. Br J Dermatol 166: 306–316. 21929537 10.1111/j.1365-2133.2011.10637.x

[37] Epstein SP, Chen D, Asbell PA (2009) Evaluation of biomarkers of inflammation in response to benzalkonium chloride on corneal and conjunctival epithelial cells. J Ocul Pharmacol Ther 25: 415–424. 19857103 10.1089/jop.2008.0140 PMC2981370

[38] Zhang EY, Chen AY, Zhu BT (2009) Mechanism of dinitrochlorobenzene-induced dermatitis in mice: role of specific antibodies in pathogenesis. PLoS One 4: e7703. 19890385 10.1371/journal.pone.0007703 PMC2766640

[39] Boislève F, Kerdine-Römer S, Rougier-Larzat N, Pallardy M (2004) Nickel and DNCB Induce CCR7 Expression on Human Dendritic Cells Through Different Signalling Pathways: Role of TNF-a and MAPK. J Invest Dermatol 123: 494–502. 15304089 10.1111/j.0022-202X.2004.23229.x

[40] Chung SH, Kweon MN, Lee HK, Choi SI, Yang JY, et al (2009) Toll-like receptor 4 initiates an innate immune response to lipopolysaccharide in human conjunctival epithelial cells. Exp Eye Res 88: 49–56. 18951893 10.1016/j.exer.2008.09.017

[41] Schultz CL, Morck DW, McKay SG, Olson ME, Buret A (1997) Lipopolysaccharide induced acute red eye and corneal ulcers. Exp Eye Res 64: 3–9. 9093015 10.1006/exer.1996.0190

[42] Dinarello CA (1991) Inflammatory cytokines: interleukin-1 and tumor necrosis factor as effector molecules in autoimmune diseases. Curr Opin Immunol 3: 941–948. 1665333 10.1016/s0952-7915(05)80018-4

[43] Dekaris I, Zhu SN, Dana MR (1999) TNF-{alpha} regulates corneal Langerhans cell migration. J Immunol 162: 4235–4239. 10201952

[44] Buron N, Micheau O, Cathelin S, Lafontaine PO, Creuzot-Garcher C, et al (2006) Differential mechanisms of conjunctival cell death induction by ultraviolet irradiation and benzalkonium chloride. Invest Ophthalmol Vis Sci 47: 4221–4230. 17003409 10.1167/iovs.05-1460

[45] Stewart M, Thiel M, Hogg N (1995) Leukocyte integrins. Curr Opin Cell Biol 7: 690–696. 8573344 10.1016/0955-0674(95)80111-1

[46] Liang H, Baudouin C, Labbe A, Riancho L, Brignole-Baudouin F (2012) Conjunctiva-associated lymphoid tissue (CALT) reactions to antiglaucoma prostaglandins with or without BAK-preservative in rabbit acute toxicity study. PLoS One 7: e33913. 22442734 10.1371/journal.pone.0033913 PMC3307783

[47] Helin M, Rönkkö S, Puustjärvi T, Teräsvirta M, Ollikainen M, et al (2011) Conjunctival inflammatory cells and their predictive role for deep sclerectomy in primary open-angle glaucoma and exfoliation glaucoma. J Glaucoma 20: 172–178. 20577105 10.1097/IJG.0b013e3181d9ccb0

